# Invited Commentary: Management of Hypergranulation Requires a Multimodal Approach

**DOI:** 10.1007/s00268-023-07227-7

**Published:** 2023-11-25

**Authors:** Daan den Hollander

**Affiliations:** University of KwaZulu Natal, Durban, South Africa

Hypergranulation—defined as abundant immature granulation tissue that grows beyond the level of the surrounding skin and is associated with a failure of re-epithelialization [1]—is a common complication of burn wounds, especially in low-resource settings, where patients often present late to a burn surgeon. Nevertheless, little evidence informs the understanding and management of this condition. A number of risk factors have been identified: healing by secondary intention, inflammation as a result of high bioburdens in the wound, hypoxia associated with occlusive dressings, excessive exudate and friction [1, 2]. However, how these factors cause a derangement of wound healing is not yet clear. Oft-repeated statements, such as that the protruding tissue provides a ’physical barrier’ to the advancing epithelium just sound too simplistic.

The management of a condition should be informed by an understanding of its etiology. The pathophysiology of wound healing is a process that is still very much under investigation. The proliferative phase, of which hypergranulation is an aberration, is initiated with macrophages synthesizing growth factors, such as Vascular Endothelial Growth Factor (VEGF) and Fibroblast Growth Factor (FGF), which recruit endothelial cells and fibroblasts to the wound and set in motion neovascularization and fibroplasia, which together establish granulation tissue [3]. Although it was originally thought that the fibroblast was the primary cell ‘orchestrating’ the formation of granulation tissue, recent work has rather revealed a picture of intensive ‘cross-talk’ between macrophages, fibroblast, endothelial cells, and epidermal cells. The role of the endothelial cells in this network has been increasingly recognized. VEGF released from epidermal cells, as well as hypoxia-induced growth factors, create gradients which direct the growth of the new vessels. VEGF-generated vessels, however, are immature, leaky and provide a low amount of oxygen and nutrients, but they are generated in abundance. When ‘sufficient’ granulation has been produced, the pro-angiogenic response including VEGF is switched off, and is followed by an anti-angiogenic response, resulting in culling of most of the new vessels through apoptosis, and maturation of the remaining vessels. Such anti-angiogenetic modulators have been identified [4]. What is not known, however, is how the system recognizes that ‘enough is enough’, how the switch from pro- to anti-angiogenic modulators is effected, and how this switch fails in the pathogenesis of hypergranulation. It has, however, been established that hypoxia and ongoing inflammation are associated with increased neovascularization and chronic wound formation.

This issue of WJS includes an article on a novel method of debridement of granulation tissue prior to grafting of burn wounds [5]. It is important to place this study in context. The removal of (hyper) granulation tissue prior to skin grafting has been a surgical dogma until the 1990s, after which the issue has been downplayed in plastic surgical textbooks [6]. Dhar et al. [6] performed a comparative study of 51 burn patients, 38 of whom had wounds on both sides of the body. All wounds on the right side were grafted after removal of granulation tissue, while all wounds of the left side were grafted directly onto the granulation tissue. They found no differences in bacteriology, graft take, and cosmetic results after 3 months. The study, however, has serious methodological issues, not least of which was the lack of a definition of ‘granulation tissue’ so that it is not clear whether they removed pathological hypergranulation tissue or physiological granulation tissue.

Hypergranulation is one component of the chronic wound spectrum along with epithelialization arrest and excessive scar formation. What the above discussion of the pathophysiology of hypergranulation has demonstrated, is that merely removal of excess tissue, albeit a necessary element in its management is not sufficient to turn the scales. Most recent discussions of the management of hypergranulation tissue have therefore stressed the need for a multimodal approach, including [2, 7]:

1. Control of infection and biofilms, such as silver, polyhexamethylene biguanide (PHMB) or medicinal honey. The use of steroids has been suggested as a second-line treatment.
2. Control of moisture and avoidance of occlusive dressings. Most authors recommend the use of a foam dressing to achieve these goals.
3. Debridement. Debridement is an important step in the management of the chronic wound, to reduce bioburden, remove senescent cells and change the wound environment from a chronic to an acute state. The use of silver nitrate sticks, popular in the past, is no longer recommended as it causes burns of the wound and may damage the underlying skin. Betaine (formulated with PHMB) and honey may provide some measure of debridement, but severe cases may require surgical debridement in theatre.
4. Prevention of friction, such as due to footwear, parts of the bed or pillows.

When managing patients with hypergranulation, it is important to keep in mind that skin tumours may present with a similar clinical picture, and to exclude these histologically.
